# Implications of climate change on the distribution of the tick vector *Ixodes scapularis* and risk for Lyme disease in the Texas-Mexico transboundary region

**DOI:** 10.1186/1756-3305-7-199

**Published:** 2014-04-25

**Authors:** Teresa P Feria-Arroyo, Ivan Castro-Arellano, Guadalupe Gordillo-Perez, Ana L Cavazos, Margarita Vargas-Sandoval, Abha Grover, Javier Torres, Raul F Medina, Adalberto A Pérez de León, Maria D Esteve-Gassent

**Affiliations:** 1 Department of Biology, The University of Texas-Pan American, Edinburg, TX 78539, USA; 2 Department of Biology, College of Science and Engineering, Texas State University, San Marcos, TX 78666, USA; 3 Unidad de Investigación en Enfermedades Infecciosas, Centro Médico Nacional SXXI, IMSS, Distrito Federal 06720, Mexico; 4 Facultad de Agrobiología, Universidad Michoacana de San Nicolás de Hidalgo, Uruapan, 60090 Michoacan, Mexico; 5 Department of Veterinary Pathobiology, College of Veterinary Medicine and Biomedical Sciences, Texas A&M University, College Station, TX 77843, USA; 6 Department of Entomology, College of Agriculture and Life Sciences, Texas A&M University, College Station, TX 77843, USA; 7 USDA-ARS Knipling-Bushland U.S. Livestock Insects Research Laboratory, Kerrville, TX 78028, USA

**Keywords:** *Ixodes scapularis*, *Borrelia burgdorferi*, Transboundary disease, Lyme disease risk map, Climate change

## Abstract

**Background:**

Disease risk maps are important tools that help ascertain the likelihood of exposure to specific infectious agents. Understanding how climate change may affect the suitability of habitats for ticks will improve the accuracy of risk maps of tick-borne pathogen transmission in humans and domestic animal populations. Lyme disease (LD) is the most prevalent arthropod borne disease in the US and Europe. The bacterium *Borrelia burgdorferi* causes LD and it is transmitted to humans and other mammalian hosts through the bite of infected *Ixodes* ticks. LD risk maps in the transboundary region between the U.S. and Mexico are lacking. Moreover, none of the published studies that evaluated the effect of climate change in the spatial and temporal distribution of *I. scapularis* have focused on this region.

**Methods:**

The area of study included Texas and a portion of northeast Mexico. This area is referred herein as the Texas-Mexico transboundary region. Tick samples were obtained from various vertebrate hosts in the region under study. Ticks identified as *I. scapularis* were processed to obtain DNA and to determine if they were infected with *B. burgdorferi* using PCR. A maximum entropy approach (MAXENT) was used to forecast the present and future (2050) distribution of *B. burgdorferi*-infected *I. scapularis* in the Texas-Mexico transboundary region by correlating geographic data with climatic variables.

**Results:**

Of the 1235 tick samples collected, 109 were identified as *I. scapularis*. Infection with *B. burgdorferi* was detected in 45% of the *I. scapularis* ticks collected. The model presented here indicates a wide distribution for *I. scapularis*, with higher probability of occurrence along the Gulf of Mexico coast. Results of the modeling approach applied predict that habitat suitable for the distribution of *I. scapularis* in the Texas-Mexico transboundary region will remain relatively stable until 2050.

**Conclusions:**

The Texas-Mexico transboundary region appears to be part of a continuum in the pathogenic landscape of LD. Forecasting based on climate trends provides a tool to adapt strategies in the near future to mitigate the impact of LD related to its distribution and risk for transmission to human populations in the Mexico-US transboundary region.

## Background

Globally, Lyme disease (LD), also known as Lyme borreliosis is considered an emerging transboundary zoonotic tick borne disease [1-7]. Foci of LD exist in the U.S, Europe, and Asia. In the US LD is the most prevalent arthropod borne infection with 30,831 cases reported to the Centers for Disease Control and Prevention (CDC) in 2012 [8] and it is estimated that over 300,000 people become infected every year [9]. The increase in LD cases during the last decade has prompted its classification as an emerging infectious disease. Several hard tick species in the genus *Ixodes* are recognized as common vectors of the spirochete *Borrelia burgdorferi*, the causative agent of LD. *Ixodes scapularis* and *I. pacificus* are the known competent vectors in the US while *I. persulcatus* and *I. ricinus* are the documented vectors in Eurasia [3,10-13]. The majority of confirmed LD cases in the US are reported from the Northeast and upper-Midwest regions [8]. Consequently, almost all LD studies done to date are centered in the Northeast and Midwest regions of the US where this disease is more prevalent, with no major studies done in Southern US (SUS). Moreover, reports of LD in Mexico continue to increase [14-16]. In this geographic region *I. scapularis* is also considered as the main vector of *B. burgdorferi*, although the involvement of other hard tick species as competent vectors is suspected [17].

Similar to other arthropod borne diseases, LD is a complex system subject to shifts in ecological processes that influence vector biology and the epidemiology of *B. burgdorferi* infection in reservoir hosts and humans [18-22]. Molecular plasticity is a feature that allows the adaptation of *B. burgdorferi* to invertebrate and vertebrate hosts where it grows [23,24]. The spirochete is maintained in the environment by different vertebrate hosts with varying degrees of competence. In the forests of eastern North America the white-footed mouse, *Peromyscus leucopus*, is its main reservoir [25,26]. On the other hand, white-tailed deer (*Odocoileus virginianus,* WTD) are the primary reproductive host for *I. scapularis* in the US, but they are reservoir-incompetent for *B. burgdorferi*[19,27]. A number of studies have suggested that vertebrate species diversity affects the risk of contracting human LD [28-32]. In addition, climate change has been predicted to affect the geographic distribution of tick vectors [33-39] and therefore the risk of LD infection. Species distribution models (SDMs) based on machine-learning algorithms and Geographic Information Systems (GIS) platforms have been used to predict areas of potential distribution of insects that serve as vectors of zoonotic infectious agents such as *Trypanosoma cruzi,* a parasite that causes Chagas disease [40,41]. These analyses typically show that climatic factors significantly influence the potential geographic distributions of vectors and reservoirs. Additionally, temperature may have a strong influence on vector species behavior [42,43]. For example, in the case of triatomine insects, temperatures higher than 30°C and low humidity increase triatomine feeding rates helping them to avoid dehydration. In addition, these insect vectors may develop shorter life cycles and higher population densities in domestic life cycles when indoor temperature increases [42]. High temperatures can also speed up the development of *T. cruzi* in vectors [44]. In regards to arthropod tick vectors, it has been observed that the spread of the tick vector *I. scapularis* in North American and Canada is followed by an increased invasion of the bacterial pathogen *B. burgdorferi* and therefore an increase in the risk of disease transmission [45]. In addition, recent studies in Canada have shown that the distribution of *I. scapularis* has been significantly affected by climate, and in particular by temperature [46,47]. Populations of this tick vector have been established and are spreading north. In this report, authors forecast, that with a continuous increase of temperature, the basic reproductive number (*R*_*0*_) of *I. scapularis* in this region is predicted to increase, and therefore the risk for Lyme disease [46].

The limited number of ecological studies in other regions like the Southern US and Gulf Coast has prevented a comparative analysis of ecological factors driving the pathogenic landscape of LD in the US [48]. In some parts of the world the ecology and epidemiology of LD remain to be fully understood. For instance, a LD-like syndrome has been described in Brazil [49], and ticks other than *I. scapularis* have been associated with the transmission of *B. burgdorferi* in Mexico [17]. Thus, LD is considered a transboundary zoonotic disease in that it can reach epidemic proportions in different regions of the globe regardless of political borders [6]. There is unequally distributed knowledge about the ecology of this disease among the regions in which it occurs, even within the US.

Knowledge gaps in our understanding of the epidemiology of LD in the Southern US exist [50]. A recent survey could not detect *B. burgdorferi* from *I. scapularis* ticks collected from deer in Tennessee [51]. During the late 1980’s and early 1990’s *B. burgdorferi* was identified in Texas using classical culturing techniques and immunofluorescence of tick midguts utilizing monoclonal anti-OspA antibody with an incidence as low as 1% [52-55]. With more advanced molecular techniques the incidence of *B. burgdorferi* in ticks collected from human subjects over a 4-year period was also near 1% [13]. However, this last study was based on a passive surveillance set up, in which ticks coming from humans were collected, and no active sampling of ticks in the state of Texas was carried out.

Human risk for infection with *B. burgdorferi* over the continental US (east of the 100^th^ meridian) has been predicted using the density of *I. scapularis* infected nymphs (DIN) [56,57]. Under this scenario southern US states were portrayed as a low risk region given the non-appearance of host seeking *I. scapularis* nymphs at sampled sites [56,57]. Worth noting is that southern states had fewer sampling sites (e.g., 9 in Texas, zero in Louisiana, 4 in Oklahoma) while other areas of the country (the northeast and midwest) were better represented (e.g. 19 in Illinois, 20 in Wisconsin, 13 in New York) [56,57]. In striking contrast to the conclusion of this purported null risk of acquiring LD in southern states, a steady number of LD cases have been reported in these low risk areas every year ( http://www.cdc.gov/lyme/stats/index.html). Other relevant epidemiological aspects that require attention include human movement because in some LD cases the disease can be acquired in a region different from the one where they are reported and differences in tick phenology and behavior at different geographic regions may exist [57]. For instance, the questing behavior of *I. scapularis* nymphs show variation among northern and southern sites, with southern nymphs rarely climbing to the top of vegetation likely reducing the ability to detect these nymphs at southern sites (J. Tsao, personal communication). These limitations might explain why the models utilized could not explain the variation in the distribution of the disease observed in low prevalence areas. Consequently, the drivers for variation in the distribution of LD cases observed in low prevalence areas remain to be identified [50].

In Mexico, a national serosurvey of human serum samples reported a *B. burgdorferi* sero-prevalence of 1.1% [15]. The Mexican states of Tamaulipas, Nuevo León, and Coahuila in the Texas-Mexico border region presented the highest sero-prevalence (6.4%) compared with the rest of the country [58]. Also, *Ixodes* ticks infected with *B. burgdorferi sensu stricto* occur in the same states [17], and the infection has been recently documented in mouse reservoirs [59] as well as in WTD [60]. Distribution models of potential tick vectors in Mexico point to a wide distribution range that overlaps not only northeastern Mexican states along the border with the US, but also extends to central Mexico [59,61]. These studies, together with confirmed clinical cases of LD, acquired in parks near Mexico City [14,62] demonstrate the existence of a enzootic cycle responsible for LD in Mexico.

The large transboundary region shared between the US and Mexico present several risk factors for zoonotic arthropod borne diseases that can be exacerbated due to climate change [6,63,64]. This situation emphasizes the need to evaluate the risk for LD from a regional standpoint. In addition, the understanding of vector borne disease ecology has improved significantly in recent years due to the advancement in molecular biology, Geographic Information Systems (GIS) and SDM [65,66].

In this study a distribution model was constructed for *I. scapularis* covering the eastern sector of the transboundary region comprising the states of Texas in the US, and Tamaulipas, Nuevo León, and Coahuila in Mexico. Future distribution models for the tick vector were forecasted under climate change scenarios within the defined study area. Additionally, data on the prevalence of *B. burgdorferi* infection in *I. scapularis* sampled at sites in Texas and the neighboring northeastern Mexican states are presented. This international research collaboration affords the opportunity to foster a binational approach for the adoption of control and prevention strategies to manage the risk, and reduce the burden of LD in human populations living along the US-Mexico border.

## Method

### Study area

**Figure 1:**
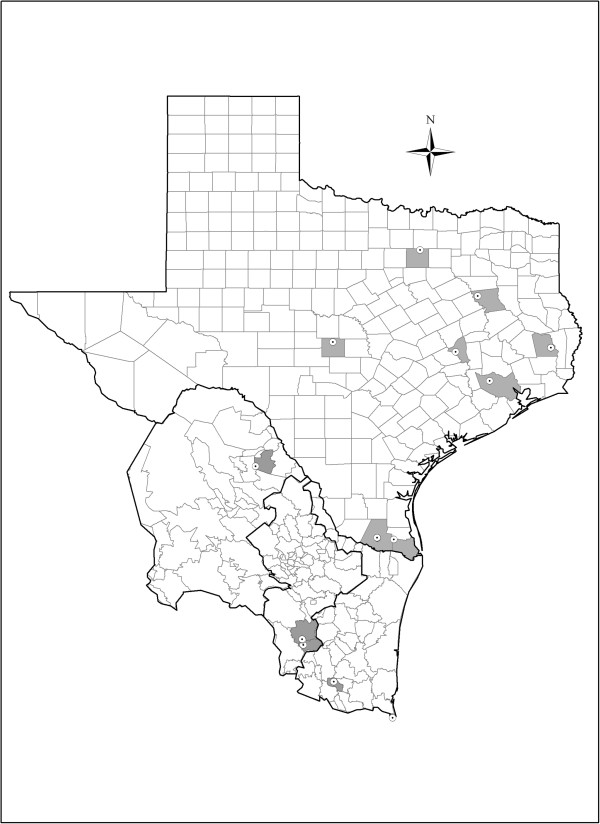
**Geographic area of study.** Each county within Texas, Tamaulipas, Nuevo León and Coahuila [72] from which we obtained *Ixodes* tick samples has been highlighted in gray. The locations of the positive samples for *B. burgdorferi* are marked with a target sign.

The Texas-Mexico transboundary region considered here includes sectors on both sides of the Rio Grande river, east of the Chihuahuan desert, which share a common biogeographic history and represents distinct ecozones. This region encompasses the Tamaulipan scrub biome. Based on the updated Köppen–Geiger climate classification [67,68], this biome climate includes four categories. The majority of the biome is classified as an arid-steppe (BSh), which changes to a temperate classification (Cfa) in Texas, whereas in Mexico it includes small areas with climate generally classified as arid desert (BWh), or temperate (Cwa). The region consists of four ecoregions including Gulf Prairies and Marshes, South Texas Plains (also known as Rio Grande Plains), Edwards Plateau (northeast of the river), and the Tamaulipan brushlands (southwest of the river) [69]. Elevation ranges from sea-level to around 1000 feet near the most inland locations. Annual rainfall may be 30-50 inches along the Gulf Coast, but declines to 14-16 inches in the inland reaches of the South Texas Plains-Edwards Plateau. Along the Gulf Coast summers are long, hot and humid, i.e. subtropical [70], and winters are very mild (>300 frost free days), while progressively more inland locations have hotter, drier summers, and winters subject to more freezing temperatures [69]. The region is subject to the fluctuations of tropical storms from the Caribbean and Gulf of Mexico, as well as periodic and sometimes extended periods of drought [71]. Within the region considered in our study we obtained and analyzed a total of 109 *I. scapularis* ticks from 16 unique geographical locations (Figure 1).

### Tick collection and classification

In Texas, ticks were collected during 2011 and 2012 from three Texas Parks and Wildlife Management Areas (WMAs) covering five counties (Anderson, Cameron, Hidalgo, Mason and Willacy), animal shelters (Brazos County), veterinary clinics from 13 counties (Bee, Collin, Edwards, Fort Bent, Harris, Kerr, Maverick, Montague, Montgomery, Robertson, Tarrant, Travis, and Uvalde), and the Big Thicket National Park in Tyler County. Counties were selected based on the presence of *I. scapularis* ticks previously reported in the literature [13,19,54-56,73]. The main sources of ticks were hunter-harvested WTD (*Odocoileus virginianus*) or gemsbok (*Oryx gazella*) brought to check stations at each WMA mentioned above. Questing ticks as well as tick samples from humans and dogs were also acquired from WMAs in Texas. Ticks collected from human subjects and their dogs at the hunter-check stations were voluntarily provided and no personal identifiers were recorded. Only location, date and the fact that the ticks were feeding on human subjects were recorded. All human subjects were properly informed about the scientific nature of the project.

For each tick specimen we gathered associated information (host species, time and date, GPS coordinates, ZIP code, County) to allow for cross-referencing our findings with the reported human LD cases in Texas. Results from those analyses will be presented somewhere else (manuscript in preparation). All ticks collected were placed in vials containing 70% ethanol and transported to the Texas A&M University College of Veterinary Medicine and Biomedical Sciences for their codification, identification using dichotomous keys [74-77], and subsequently processed for DNA extraction and *B. burgdorferi* detection.

In Mexico, *I. scapularis* ticks were collected in the northeastern states of Tamaulipas, Nuevo Leon, and Coahuila. Tick specimens were obtained from four different regions where vegetation was sampled for questing ticks and from collected wild mammals (eastern cottontail, the hispid cotton rat, the painted spiny pocket mouse, prairie dogs, ringtail, black-tailed jackrabbit and jaguar) and domestic animals (bovids, horses, and dogs) [17,78]. Ticks were preserved in 70% ethanol and archived. Two institutions were involved in the collections, the Entomology Laboratory of Escuela Nacional de Ciencias Biologicas, IPN, Mexico, which collected in forested and rural areas during 1990-1998, and the Emerging Infectious Disease Laboratory, UIMEIP, Centro Medico Nacional SXXI, IMSS, which collected ticks in forested areas during 1999-2007 [17,78]. An additional record of *I. scapularis* was obtained from the report of this species occurring on a female jaguar (*Panthera onca*) from the Reserva de la Biosfera, El Cielo in Tamaulipas Mexico [79]. All wild mammal collections were done under the approved permits by Dirección General de Vida Silvestre in Mexico.

### *Borrelia burgdorferi* identification

DNA was extracted from individual *I. scapularis* ticks collected during the sampling period described above using the High Pure PCR template preparation kit for genomic DNA (Roche, Indianapolis, IN) following manufacturer’s recommendations with modifications to adapt the protocol to ticks. Briefly, individual ticks were homogenized utilizing the bead mill BeadRuptor 24 (Omni International, Inc., Kennesaw, GA) in 200 μl of Phosphate Buffer Saline (PBS) plus 200 μl of lysis buffer and 800 μg of Proteinase K in 2 ml screw cap tubes for 5 minutes at a 5.65 m/s intensity (equivalent to 210 × *g*). During this step 1.4 mm ceramic beads were used to homogenize unengorged ticks while 2.8 mm ceramic beads were used with engorged ticks. After homogenizing, each sample was centrifuged at 10,000 × *g* to pellet debris. Supernatants were collected and 200 μl of binding buffer was added to each sample and the mixture incubated at 70°C for 10 minutes followed by the addition of 100 μl of isopropanol. The mixture was run through filter columns at 8,000 × *g* for 1 minute. DNA bound to the filter was washed and eluted following manufacturer recommendations. Eluted DNA concentration and purity were measured using a NanoDrop (Thermo Scientific, Inc., Willinton, DE). DNA was used for the detection of *B. burgdorferi sensu lato* by PCR using the flagella gene (*flaB*) as previously described by Jaulhac *et al*. [12]. Amplicons were separated on a 1.5% agarose gel and positive bands were sent for sequencing (Eton Biosciences, San Diego, CA). Sequences were analyzed through BLAST® using MacVector 12.6 software (MacVector Inc., Cary, NC) to facilitate species and strain identification. A second nested PCR for the intergenic region (IGR) 16SrRNA-23SrRNA using the primers and conditions described by Bunikis *et al.*[23] was done to all *flaB* positive samples with *B. burgdorferi* identification. IGR amplicons were also submitted for sequencing and results analyzed using the MacVector 12.6 software to facilitate strain identification. Water served as negative control, and genomic DNA isolated from *B. burgdorferi* B31 A3 strain culture as positive control in all PCR steps done in this study.

### Modeling methods and evaluation

To construct the potential distribution for *I. scapularis* at the study area we compiled a database with unique locations (e.g., different geographic coordinates) of presence records that cover most of the known range of this species. Locations were obtained from the scientific literature [56] and from our own fieldwork (Texas and Northern Mexico). Our modelling methods required identification of precise locations where this tick species was found but a prevalent issue regarding tick records is that most lack an adequate level of geographical precision or reliable taxonomic identification. For example, the most extensive collection of reliable records for *I. scapularis*, covering most of its range, is limited to county level precision [80] and thus it is too rough for our purposes. Instead, we accessed data from several published studies that included standardized collecting techniques to sample 304 sites throughout 37 states from the continental US east of the 100^th^ parallel. This study consisted mostly of state parks or natural preserves [56,57,81,82]. *I. scapularis* was found at 94 of these sites [56]. This was the basis for our locations. For each park or natural area where *I. scapularis* was recorded we identified a geographical centroid that was verified to occur within natural vegetation (via Google Earth) and the corresponding geographic coordinates were used as a unique location. Since most of the parks are small (<10,000 Ha), the level of resolution is substantially better than relying on county level records.

Present and projected future potential distributions for the target species were computed using these presence records for the species (longitude/latitude) and using climatic parameters as exploratory variables, using a maximum entropy algorithm (MaxEnt) [66,83-85]. MaxEnt predicts a probability distribution ranging from 0 (least suitable) to 1 (most suitable) of habitat suitability over a given study area [84,86]. We used MaxEnt version 3.3.3 k ( http://www.cs.princeton.edu/~schapire/maxent/) with the default modeling parameters (convergence threshold = 10^5^, maximum iterations = 500, regularization value β = auto) following conditions standardized elsewhere [86]. We used the bioclimatic variables available at http://www.worldclim.org as predictors (Table 1).

**Table 1:** List of the environmental variables used in this study

**Variable**	**Description**
BIO1	Annual Mean Temperature
BIO3	Isothermality
BIO5	Maximum Temperature of Warmest Month
BIO6	Minimum Temperature of Coldest Month
BIO7	Temperature Annual Range (maximum temperature of warmest month – minimum temperature of coldest month)
BIO9	Mean Temperature of Driest Quarter
BIO10	Mean Temperature of Warmest Quarter
BIO11	Mean Temperature of Coldest Quarter
BIO12	Annual Precipitation
BIO13	Precipitation of Wettest Month
BIO14	Precipitation of Driest Month
BIO15	Precipitation Seasonality (Coefficient of Variation)
BIO16	Precipitation of Wettest Quarter
BIO17	Precipitation of Driest Quarter
BIO18	Precipitation of Warmest Quarter
BIO19	Precipitation of Coldest Quarter

**Figure 2:**
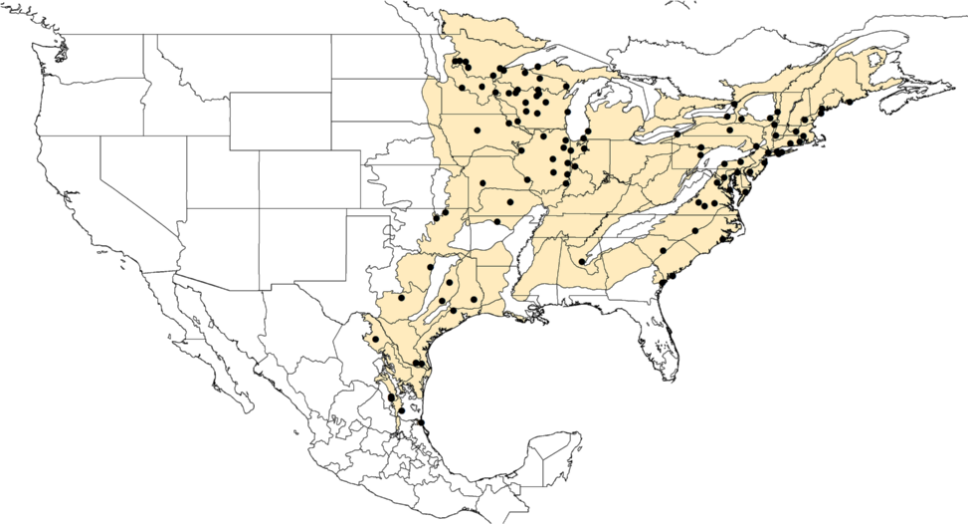
**Geographic distribution of *I. scapularis *by biogeographic regions (yellow shadow) and states in the US and Mexico.** Each locality represent a location in which *I. scapularis* was detected in this study as well as those previously published [56]. Localities are represented as black dots.

These bioclimatic variables have a resolution of approximately 1 × 1 km^2^ grid cells. To avoid the use of highly correlated variables, we ran a correlation analysis and eliminated one of the variables in each pair with a Pearson correlation value > 0.8 (Additional file 1: Table S1). Sixteen variables were used to construct the model (Table 1). To avoid the use of artifactual distribution limits for our target species (e.g., political boundaries), we used as base map to construct our models a biogeographic regions map (Figure 2) and then projected the models to Texas and Northeastern Mexico (Tamaulipas, Nuevo Leon, Coahuila) [87]. Biogeographic regions are areas of animal and plant distribution having similar or shared characteristics, thus this map is considered essential to construct a more realistic model of potential distribution for *I. scapularis*. Model results were processed and visualized using ArcGIS 10. The Area Under the Curve (AUC) of Receiver Operating Characteristics plots (ROC) was calculated to evaluate the models [88]. ROC is a threshold–independent measure that evaluates the sensitivity (probability that the model produces a positive result in a positive locality) versus the specificity (probability that the model produces a negative result in a negative locality) of a model when presented with new data. A ROC plot is obtained by plotting all sensitivity values on the *y*–axis against their equivalent (1–specificity) values for all available decision thresholds on the *x*–axis. The theoretically perfect result is AUC = 1, whereas a test performing no better than random yields AUC = 0.5. The AUC was calculated in MaxEnt.

### Projection under future scenarios

Future distribution models were developed for the year 2050 using two climate scenarios from the Intergovernmental Panel on Climate Change (IPCC): A2A and B2A. These scenarios assume a heterogeneous world, but with B2A being more environmentally focused than A2A. B2 describes a world in which the emphasis is on local solutions to economic, social, and environmental sustainability. It is a prospective view of the world with continuously increasing global population, at a rate lower than A2, and intermediate levels of economic development [43]. Different Global Circulation Models (GCM) exist, and since no single one can be considered superior, we selected three different GCM to compare results for potential distributions: the Canadian Centre for Climate Modeling and Analysis (CCCMA), the Commonwealth Scientific and Industrial Research Organization (CSIRO) and the Hadley Centre for Climate Change (HADCM3) models.

We estimated a “stable” suitable habitat considering present and future maps (both IPCC scenarios and the three GCM). First, we reclassified the potential distribution models for the present and future to obtain binary maps (presence = 1, absence = 0) using the “minimum presence threshold”. This is the most conservative threshold that includes suitable habitat predictions for all the locations where we have collected *I. scapularis* ticks. The binary maps were then projected onto UTM with a spatial resolution of 1 km^2^. The “stable” habitat was estimated as the area that remained constant under each scenario and GCM.

## Results

During this study, a total of 1235 tick specimens were collected in the Texas-México transboundary region. One hundred and nine specimens (8.83%) were identified as *I. scapularis,* which were collected questing or from WTD, dogs, gemsbok, and a cat in Texas, and eastern cottontail (*Silvilagus floridanus*), painted spiny pocket mouse (*Lyomis pictus*) and jaguar (Pantera Onca) in Mexico, as shown in Table 2. Forty five percent of the *I. scapularis* were infected with *B. burgdorferi* by PCR and sequencing of the flagella and IGR amplicons.

**Table 2:** **Texas counties and Mexico districts from which *I. scapularis *** **ticks were collected**

	**State**	**County or District**	***Localities***	**Ticks collected**	***I. scapularis *** **(%)**^**#**^	***I. scapularis *** infected (%)**	**Host**^⌘^
*US*	Texas	Anderson	1	64	26/64 (40.6)	13/26 (50.0)	WTD (13)
	Texas	Brazos	1	45	5/45 (11.1)	3/5 (60.0)	Dog (2)
							WTD (1)
	Texas	Cameron	1	32	3/32 (9.4)	2/3 (66.7)	WTD (2)
	Texas	Fort Bent	1	65	1/65 (1.5)	1/1 (100.0)	Dog (1)
	Texas	Hidalgo	2	7	1/7 (14.3)	0/1 (0.0)	Dog
	Texas	Mason	1	148	5/148 (3.4)	2/5 (40.0)	Oryx (2)
	Texas	Tarrant	1	4	3/4 (75.0)	2/3 (66.7)	Cat (2)
	Texas	Tyler	1	37	29/37 (78.4)	13/29 (44.8)	Questing (13)
	TOTAL		9	574	74/574 (12.9)	37/74^ (50.0)	
*Mexico*	Nuevo Leon	San Josesito, Zaragoza	*3*	230	31/230 (13.5)	8/25 (29)	*Sylvilagus floridanus*
			*1*			1/6	*Lyomis pictus*
	Tamaulipas	Tampico	*1*	51	2/51 (3.9)	2/2 (100)	Vegetation
	Tamaulipas	El Cielo, Gomez Farias	*1*	379	1/379 (0.02)	1/1 (100)	*Panthera onca*
	Coahuila	La Rosita, San Pedro	*1*	1	1/1 (100)	0/1(0)	-
	TOTAL		*7*	661	35/661 (5.29)	12/35 (34.28)	

#: Percentage of *I. scapularis* found in each county among other tick species.

**: Percentage of *I. scapularis* infected per county.

^: 6.445% of all ticks collected were *Ixodes scapularis* infected with *Borrelia burgdorferi*.

⌘: in parenthesis is represented the numbers of infected *Ixodes scapularis* ticks isolated from each host.

### Presence of *I. scapularis* and *B. burgdorferi* infection in the Texas-Mexico transboundary region

**Figure 3:**
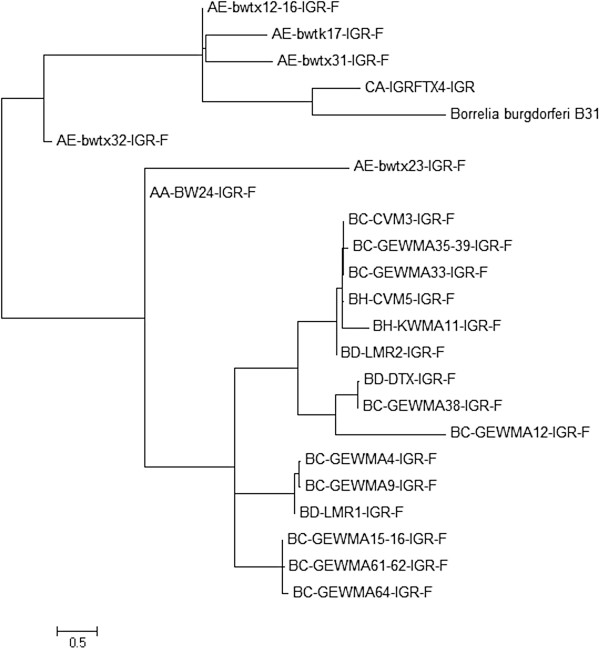
***Borrelia burgdorferi *strains detected in Texas.** For constructing the dendrogram the IGR sequences were analyzed using MEGA 5.2 (Molecular Evolutionary Genetics Analysis, http://www.megasoftware.net/). A phylogenetic reconstruction analysis was obtained through maximum likelihood using the Tamura–Nei nucleotide substitution model.

A total of 574 tick specimens were collected from twenty counties across the state of Texas. Of those, 74 (12.89%) *I. scapularis* ticks were collected in eight of the counties sampled (Table 2). Fifty percent of *I. scapularis* ticks showed infection with *B. burgdorferi sensu stricto* (Table 2). This accounts for 6.445% of the total number of ticks tested in Texas in the current study. Even though the majority of the *B. burgdorferi* infected *I. scapularis* ticks were found on the most Eastern counties in Texas (Anderson and Tyler Counties), they were also detected in Central (Mason County), and South Texas (Cameron County, located on the border with Mexico). The central and south Texas areas had been previously considered as unsuitable for *I. scapularis*[56,65,89]. Only one location in Texas (Hidalgo, Table 2) showed no positive *I. scapularis* ticks for *B. burgdorferi*. When analyzing the intergenic region 16S-23S rRNA of *B. burgdorferi* detected in the ticks collected in Texas, the sequences tend to group in two main clusters (Figure 3). These results suggest that strains similar to the positive control used in this study, *B. burgdorferi* B31 strain, were rarely detected in the area of study.

Of the 661 ticks collected from 11 localities in México, 203 were identified as *Ixodes* sp and only 35 as *I. scapularis*. Twelve *I. scapularis* were infected with *B. burgdorferi* and were collected in six of the 7 localities (Table 2). The infected *Ixodes* ticks were predominantly from four rural localities in San Josesito district, state of Nuevo Leon, and only one locality in Tampico district, state of Tamaulipas. A single *I. scapularis* tick was collected in Coahuila, which was not infected with *B. burgdorferi*.

### Present and future suitable habitat for *I. scapularis* in the Texas-Mexico region

**Figure 4:**
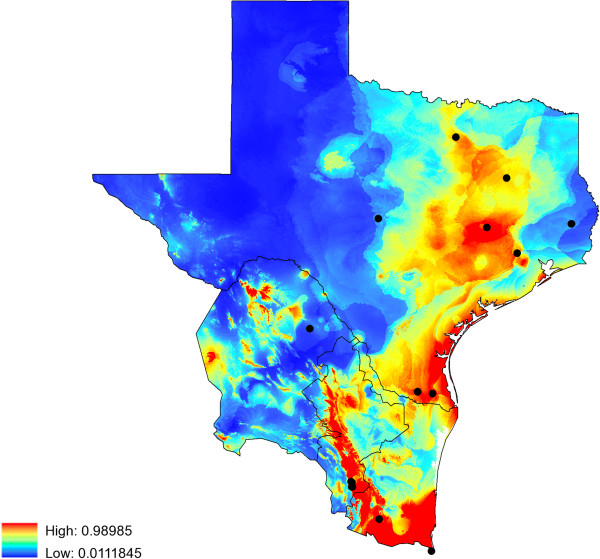
**Present suitable habitat for *Ixodes scapularis *obtained with a maximum-entropy approach using the localities recorded of infected and non-infected *I. scapulairs *ticks (black dots) considering 17 climatic variables (temperature-precipitation).** Red = high suitable habitat vs. blue = no suitable habitat for *I. scapularis*.

**Figure 5:**
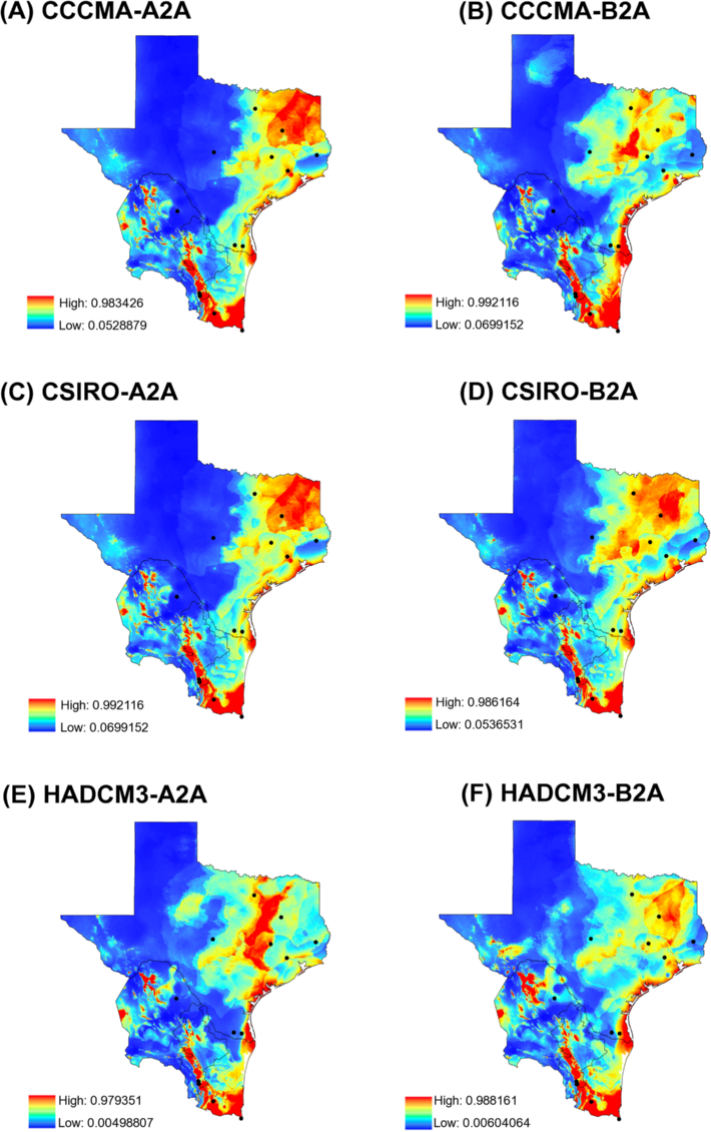
**Future (year 2050) suitable habitat for *Ixodes scapularis *obtained with a maximum-entropy approach. (A-F)** potential distributions for *I. scapularis*. Black dots = geographic locations of infected and non-infected ticks. Red = high suitable habitat vs. blue = no suitable habitat for *I. scapularis*. General circulatory models and climatic scenarios: **(A)** CCCMA-A2A; **(B)** CCCMA-BA2; **(C)** CSIRO-A2A; **(D)** CSIRO-BA2; **(E)** HADCM3-A2A; **(F)** HADCM3-B2A. Applied to general circulatory models and climatic scenarios.

One hundred and ten unique locations, at which *I. scapularis* is already present, were used to develop models of present and future suitable habitats for *I. scapularis* (Figure 2). From those, 94 came from the literature and 16 from our fieldwork: 9 were from Texas, and 7 were from NE Mexico states along the Texas-Mexico border (Table 2). The model of potential distribution for *I. scapularis* had an AUC =0.831. Comparison of present (Figure 4) and future (Figure 5A-F) suitable habitat shows potential shifts in the distribution of suitable habitat for *I. scapularis* due to climate change. As shown in Figure 4, the area of *I. scapularis* distribution is expected to expand in Northeastern Texas as well as along the coastal areas, whereas in Mexico the model predicts maintenance of the distribution area in the three northeastern states. Interestingly, there is a continuous line along the gulf coast that will remain a highly suitable habitat for this tick vector in all of the forecasting maps generated (Figure 5A-F).

**Figure 6:**
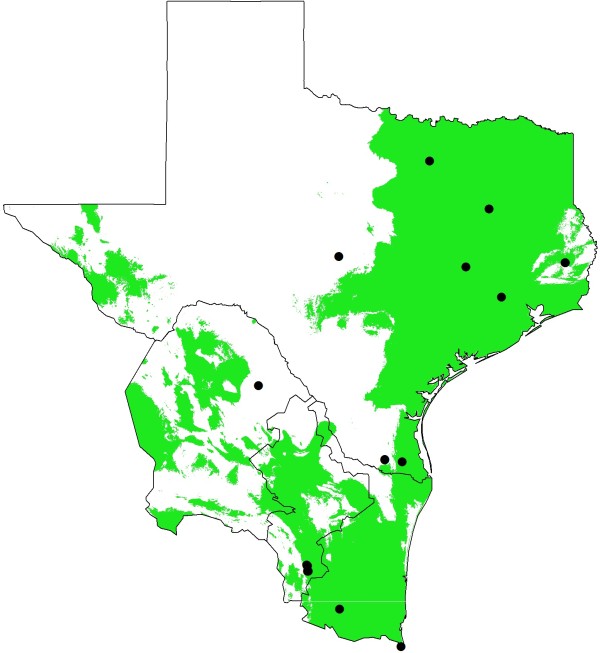
**Stable area for the distribution of *I. scapularis *in the geographic area of study.** Highlighted is the area in the Texas-Mexico region that will remain suitable for the maintenance of *Ixodes scapularis* populations present and future (2050) regardless of the prediction made (IPCC scenario and GCM). The total area is estimated in 569,910 Km^2^. Black dots denote the localities we recorded for infected and non-infected *I. scapularis* ticks.

The climatic variables that contributed the most to the model are presented in Table 3. Briefly, isothermality accounts for 20% together with precipitation of the wettest quarter (18%), the maximum temperature of the warmest month (14.6%), and the precipitation observed in the wettest month (11.5%). These parameters account for 64.2% of the average model. The correlation between each one of these four relevant environmental variables and the probability for the presence of *I. scapularis* confirm the close tie of this species to precipitation and temperature. Considering these environmental parameters, the suitable area for the survival of *I. scapularis* that will be maintained stable regardless of the forecasting model used will cover an area of 569,910 Km^2^ along Eastern Texas all the way to Northern Mexico and along the Gulf Coast (Figure 6).

**Table 3:** Environmental variables mostly affecting the model developed in this study

**Variable**	**Percent contribution**
Isothermality (Mean Diurnal Range/Temperature Annual Range) × 100	20.0
Precipitation of Wettest Quarter	18.1
Max Temperature of Warmest Month	14.6
Precipitation of Wettest Month	11.5

## Discussion

As an emerging transboundary zoonotic tick-borne disease, LD affects thousands of humans and domestic animals around the world [5,7,90]. Transboundary zoonotic diseases can maintain a dynamic focus and have pathogens circulating in a geographic region encompassing multiple geopolitical boundaries [64]. The sector of the US-Mexico border shared by south Texas and the states of Tamaulipas, Nuevo León, and Coahuila, is inhabited by millions of people and includes a biogeographic region linking the Neotropic and Nearctic ecozones where foci of vector-borne diseases like LD exist [91-94]. The distribution map presented here documents the transboundary aspect for the risk of contracting LD in the region encompassing Texas and northeast Mexico by taking into account the presence of the tick vector *I. scapularis* infected with *B. burgdorferi*.

There are a number of studies evaluating the distribution of *I. scapularis*, the competent vector for the transmission of LD [28,47,51,56,57,82,95-98], and how its distribution affects the risk for human disease. The analysis in most of these studies is focused in the Northeastern and Midwest US as well as in Canada. A climate based model classified each of 3,628 0.5° grid cells, covering the US, as places where established, suitable and unsuitable conditions to support *I. scapularis* exist, thus providing an estimation of its geographic distribution in the US [89]. This model portrays most of East Texas as suitable habitats or where established populations can exist [89]. Our current species distribution model of *I. scapularis* overlaps greatly over East Texas with the climate-based model but also expands the range of habitat identified as suitable for *I. scapularis* towards Central and South Texas through the Gulf Coast. Additionally, our model expands into northern Mexico as a continuous geographic landmark to maintain a stable *I. scapularis* population at this border region. Visualization of the predicted suitable area depicted in Figure 5 is further evidence that the Texas-Mexico transboundary region is part of a continuum in the pathogenic landscape of LD serving as a corridor linking *B. burgdorferi* with tick vectors and mammalian reservoirs in the Neotropic and Nearctic ecozones. It remains to be determined if migratory birds are the only animals dispersing infected *I. scapularis* in the region, which could establish LD foci [99]. More detailed molecular studies of *Borrelia* spp. identified in *I. scapularis* and other ticks will enhance our understating of the diversity among species in the *B. burgdorferi sensu lato* complex and the tick vectors involved in the epidemiology of Lyme borreliosis in the Texas-Mexico transboundary region [5,61,100].

No specific distribution model exists for *I. scapularis* in Mexico but a distribution model for the genus *Ixodes*, generated with similar methodologies to our model, also predicts a wide distribution covering northeastern Mexico [61]. Although our model also overlaps greatly with this genus level model, especially over the mountainous regions of Tamaulipas, Nuevo Leon, and Coahuila, it expands the distribution of *Ixodes* over the coastal plain of Tamaulipas thus connecting to the range in Texas along the Gulf Coast area. The level of resolution of our model is substantially greater than in previous climate based models [89,101] because of the smaller grid cell used (1 Km × 1 Km cells in our model versus roughly 55 × 42 Km cells), thus allowing a more precise prediction of sites where *I. scapularis* currently occurs. To our knowledge, this report is the first attempt to model how the range of *I. scapularis* in the Texas-Mexico transboundary region will be affected under specific climatic change scenarios. Only one location in Texas showed no positive *I. scapularis* ticks for *B. burgdorferi*. We attribute this to the low number of ticks (only 7) collected with only one *I. scapularis*. Geographical areas in which a species occur are determined by several ecological, evolutionary, and historical factors. These factors can be related to (1) abiotic aspects (e.g., elevation, climate); 2) biotic aspects (i.e., species interactions like competition and parasitism); 3) historical characteristics (e.g., barriers, speciation process); 4) dispersal capabilities; 5) accessible regions for dispersal; 6) evolutionary capability of a species to adapt to new conditions; and 7) anthropogenic influences (e.g., changes in land use, translocation of organisms; [102,103]). The most frequently used factors to estimate species distributions are climatic variables [104,105]. Climate can limit the distribution of animals directly by affecting growth or survival (e.g., lower and upper lethal temperatures), and indirectly via interacting species (e.g., food sources, pathogens, competitors, or predators). While testing the model, two locations in the region under study, had low values of suitable habitat predicted by MaxEnt for *I. scapularis*. These two localities are in West Texas (Mason County) and in the Mexican state of Coahuila. These are two of the most western locations, sampled, where *I. scapularis* was detected in vegetation and feeding on exotic ungulates inhabiting gated properties. Consequently, these locations are considered of great biological relevance, because they could represent the western boundary of the geographic region suitable for *I. scapularis*. This observation highlights the need for ecological studies to understand the phenology and host preference in locations marginally suitable for the blacklegged tick.

Although our species distribution model is congruent with the spatial distribution of LD cases in humans for Texas (see maps in http://www.cdc.gov/lyme/stats/index.html), a direct correlation cannot be established because additional vector density and *B. burgdorferi* prevalence data need to be gathered, and human movement has epidemiological consequences since infection can be acquired in a region different from the one where it is reported [27]. The density of infected *I. scapularis* nymphs (DIN) has been suggested as a strong predictor for LD risk of infection [56,57,82,89,101]. For Texas this risk of infection has been deemed low because no *I. scapularis* nymphs were detected using standardized sampling techniques over nine localities in the state [56,82,106]. This contrasts with the continuous reports of human LD cases and the detection of canine LD in the state [107-109]. As an initial approach to understand where infected *I. scapularis* ticks occurred in Texas and Mexico, we performed a combination of passive and active searches of different developmental stages of this competent LD vector. In our search we did find *B. burgdorferi* infected adult *I. scapularis* ticks in areas were human LD has been reported [8] and where canine LD has been observed [108]. The prevalence of *I. scapularis* infected with *B. burgdorferi* detected was higher than the 1% recorded in the early 1990’s using classical culture and microscopy techniques for the detection of *B. burgdorferi*[52,54,55], and than that observed more recently through a passive surveillance study and molecular techniques [13]. The adult ticks found in this preliminary study were either questing on vegetation or feeding on wildlife (WTD and gemsbok) and companion animals (mainly dogs). Therefore, our results suggest that infected *I. scapularis* nymphs are present in Texas, but sampling methods standardized for the conditions in Northeast and Midwest US sites might not be optimal to sample a representative *I. scapularis* nymph population questing in the Texas-Mexico transboundary region. Variation in environmental conditions influencing questing behavior may significantly impact the type of host ticks encounter and may lead to differential host use within a particular study area [110-113]. Therefore, further studies testing different sampling procedures, including different times of the day and seasons are currently being developed by our team. Our goal is to determine the phenology of *I. scapularis* in the Southern US and Mexico and the questing behavior of the different developmental stages to determine how that will affect the risk for LD in humans and companion animals.

In forested regions of the northeastern US a strong determinant of nymph density, and percentage of *Borrelia* infected nymphs, is the composition of the mammalian assemblage present at a given area [25,26]. At locales where the main competent reservoir, *P. leucopus*, is the dominant component of the assemblage, a higher prevalence of *Borrelia* infected nymphs is present, and that prevalence diminishes as other small mammal species become more common [25,26]. *P. leucopus* has a wide distribution range from Canada to south Mexico and although it is the most common small mammal in mixed forests (deciduous and coniferous) at eastern states it can be more restricted in the western and southern parts of its range [114]. This rodent uses diverse habitats and the plant communities it inhabits in the northeastern US are strikingly different from the more xeric habitats in the south, to the point that it is rarely found in forests but is more common in highly disturbed habitats in Mexico [114]. Given these differences of habitat use between northern and southern localities, the relative abundance of *P. leucopus* may vary between different regions. Assessing host range for *I. scapularis* nymphs and the reservoir competence for *B. burgdorferi* of other small mammals is needed to understand the pathogenic landscape of LD in the Texas-Mexico transboundary region.

The tick collection effort reported here was initiated in Texas during a historical drought in the Southern US ( http://droughtmonitor.unl.edu), with record high temperatures and record low precipitations affecting 100% of the Texas with “abnormally dry” to “severely dry” conditions that continued in some parts of the state until 2013. Even under these conditions we were able to collect *I. scapularis* ticks. This observation suggests that there were areas where microhabitat conditions allowed blacklegged ticks to overcome high temperatures and desiccation. Under this scenario continuous sampling of our area of study will be essential to determine where and how the immature stages of *I. scapularis* survive. In addition, continuous sampling of more localities will help validate and refine the generated maps that forecast the current and future distribution of this tick vector in the Texas-México transboundary region. However, public safety issues in northern Mexico have prevented further fieldwork. Current efforts are focused on additional sampling of Texas locations utilizing different sampling techniques to enhance detection of *I. scapularis* nymphs. Ongoing samplings of small mammal assemblages in Texas will help us understand which are the vertebrate hosts for the immature stages of *I. scapularis* in Southern latitudes.

In our analyses isothermality, maximum temperature of the warmest month, precipitation of the wettest month, and the precipitation of the wettest quarter were the environmental factors that contributed the most to the model. The correlation between each one of these four relevant environmental variables and the probability for the presence of *I. scapularis* confirm the close tie of this species to precipitation and temperature [58,62]. These results correspond with the fact that *I. scapularis* survival depends on the temperature and humidity of a particular region [18,36,115]. Moreover, average precipitation in a certain area can explain the fluctuation of LD cases reported to CDC. The total number of LD cases reported appears to follow the 2-year tick density pattern, in which a year with more rain and higher number of ticks are followed by a peak in LD cases two years later [18]. Taken together, our results suggest that similar parameters to those observed in other parts of the country govern the distribution of *I. scapularis* in Texas. However, attention is required to adapt the timing and methodologies for tick sampling campaigns to optimize the collection of the different developmental stages of this tick species. Several combinations of climatic variables were explored to generate our current model, but no major changes in the distribution of infected *I. scapularis* were observed over the region of interest. More research is required to ascertain similarities and differences in the ecology of LD between the northeast and upper-midwest parts of the US and the Texas-Mexico transboundary region and their effect on the risk of exposure to *B. burgdorferi* among human and domestic animal populations. These efforts are expected to enhance the robustness of the *I. scapularis* distribution model presented here. In combination with genetic data, consideration of the entire black legged tick range in a refined version of our model would allow testing if genetic variation of *I. scapularis* is structured according to geography, to host use, or both. Geographic and host-based structure has been documented for *I. ricinus* and other *Ixodes* species [111,112,116-118].

## Conclusion

The model presented here shows that*,* the distribution range of *I. scapularis*, the competent vector for the transmission of LD can potentially be widely occurring in Eastern Texas and Northern parts of Mexico along the Gulf Coast as a continuous region. Extreme climate change conditions predict the maintenance of suitable areas for *I. scapularis* similar to those currently observed and potentially expanding towards central Texas. The ecology of LD remains to be fully understood in the Texas-Mexico transboundary region. Under these conditions, it will be necessary to continue the study of the distribution of this tick vector, at the western and southern edges of its known range. Consequently, research on the range of hosts the larvae and nymphs of *I. scapularis* can parasitize is required to enhance our understanding of the risk for LD transmission in the Southern US and México. The enzootic cycle of *B. burgdorferi* in this region remains to be fully understood. A binational and collaborative approach to such scientific efforts is recommended to develop and adapt control and prevention strategies that will help manage the risk, and reduce the burden of LD in human populations living along the US-Mexico border.

## Competing interests

The authors declare that they have no competing interests.

## Authors’ contributions

TPF participated in the design of the study, carried out the organization of data bases, developed the distribution models presented in the paper and participated in the organization and drafting of this manuscript. ICA and APL participated in the study design, data base analysis, and in the organization and drafting of this manuscript. GGP participated in the design of the study, coordinated the sample collection in Mexico, provided the Mexican database, and participated in the organization and drafting of this manuscript. ALC carried out the data analysis and participated in the generation of distribution models presented. MVS participated in the design of the fieldwork carried out in Mexico, identified tick species and provided the distribution data. AG participated in the design of the field work carried out in Texas and collected samples, analyzed DNA by PCR, analyzed sequencing sequences and provided data for Figure 1 of this paper. JT, coordinated the analysis of Mexican samples and participated in the organization and drafting of this manuscript, RFM participated in the organization and drafting of this manuscript. MDEG participated in the design of the study, coordinated and participated in the sample collection and analysis of the Texas samples, provided the Texan database, coordinated the organization and drafting of this manuscript as well as the acquisition of funds to carry out the study. All authors read and approved the final manuscript.

## Supplementary Material

Additional file 1: Table S1 Correlations Pearson two-tiled. Click here for file
