# Supplementary material for: Implications of climate change on the distribution of the tick vector Ixodes scapularis and risk for Lyme disease in the Texas-Mexico transboundary region
Source: Parasit Vectors. 2014 Apr 25;7:199. doi: 10.1186/1756-3305-7-199 (PMC4022269; doi:10.1186/1756-3305-7-199)
Supplement: Additional file 1: Table S1 — Correlations Pearson two-tiled. [file 1756-3305-7-199-S1.docx]

Additional file 1: Table S1

| **Correlations Pearson two-tiled** | | | | | | | | | | | | | | | | | | | | |
| --- | --- | --- | --- | --- | --- | --- | --- | --- | --- | --- | --- | --- | --- | --- | --- | --- | --- | --- | --- | --- |
|  | | bio_01 | bio_02 | bio_03 | bio_04 | bio_05 | bio_06 | bio_07 | bio_08 | bio_09 | bio_10 | bio_11 | bio_12 | bio_13 | bio_14 | bio_15 | bio_16 | bio_17 | bio_18 | bio_19 |
| bio_01 | Pearson Correlation | 1 | .162 | .680^**^ | .296^**^ | .849^**^ | .967^**^ | -.827^**^ | .453^**^ | .803^**^ | .887^**^ | .976^**^ | .236^*^ | .526^**^ | .182 | .039 | .289^**^ | .225^*^ | -.050 | .286^**^ |
|  | Sig. (2-tailed) |  | .091 | .000 | .002 | .000 | .000 | .000 | .000 | .000 | .000 | .000 | .013 | .000 | .056 | .686 | .002 | .018 | .604 | .002 |
|  | N | 110 | 110 | 110 | 110 | 110 | 110 | 110 | 110 | 110 | 110 | 110 | 110 | 110 | 110 | 110 | 110 | 110 | 110 | 110 |
| bio_02 | Pearson Correlation | .162 | 1 | .566^**^ | -.277^**^ | .204^*^ | .087 | -.014 | .218^*^ | .039 | .026 | .188^*^ | -.310^**^ | -.079 | -.308^**^ | .364^**^ | -.155 | -.303^**^ | -.191^*^ | -.271^**^ |
|  | Sig. (2-tailed) | .091 |  | .000 | .003 | .033 | .365 | .885 | .022 | .683 | .788 | .050 | .001 | .414 | .001 | .000 | .106 | .001 | .046 | .004 |
|  | N | 110 | 110 | 110 | 110 | 110 | 110 | 110 | 110 | 110 | 110 | 110 | 110 | 110 | 110 | 110 | 110 | 110 | 110 | 110 |
| bio_03 | Pearson Correlation | .680^**^ | .566^**^ | 1 | .008 | .318^**^ | .747^**^ | -.801^**^ | .228^*^ | .598^**^ | .299^**^ | .792^**^ | -.050 | .422^**^ | -.067 | .329^**^ | .172 | -.055 | -.091 | .002 |
|  | Sig. (2-tailed) | .000 | .000 |  | .931 | .001 | .000 | .000 | .017 | .000 | .002 | .000 | .601 | .000 | .484 | .000 | .073 | .568 | .346 | .980 |
|  | N | 110 | 110 | 110 | 110 | 110 | 110 | 110 | 110 | 110 | 110 | 110 | 110 | 110 | 110 | 110 | 110 | 110 | 110 | 110 |
| bio_04 | Pearson Correlation | .296^**^ | -.277^**^ | .008 | 1 | .232^*^ | .374^**^ | -.367^**^ | -.213^*^ | .373^**^ | .303^**^ | .315^**^ | .589^**^ | .024 | .734^**^ | -.732^**^ | .038 | .735^**^ | -.109 | .684^**^ |
|  | Sig. (2-tailed) | .002 | .003 | .931 |  | .015 | .000 | .000 | .025 | .000 | .001 | .001 | .000 | .801 | .000 | .000 | .695 | .000 | .256 | .000 |
|  | N | 110 | 110 | 110 | 110 | 110 | 110 | 110 | 110 | 110 | 110 | 110 | 110 | 110 | 110 | 110 | 110 | 110 | 110 | 110 |
| bio_05 | Pearson Correlation | .849^**^ | .204^*^ | .318^**^ | .232^*^ | 1 | .700^**^ | -.415^**^ | .561^**^ | .563^**^ | .976^**^ | .722^**^ | .158 | .293^**^ | .098 | -.011 | .139 | .148 | -.109 | .194^*^ |
|  | Sig. (2-tailed) | .000 | .033 | .001 | .015 |  | .000 | .000 | .000 | .000 | .000 | .000 | .099 | .002 | .307 | .906 | .148 | .123 | .255 | .043 |
|  | N | 110 | 110 | 110 | 110 | 110 | 110 | 110 | 110 | 110 | 110 | 110 | 110 | 110 | 110 | 110 | 110 | 110 | 110 | 110 |
| bio_06 | Pearson Correlation | .967^**^ | .087 | .747^**^ | .374^**^ | .700^**^ | 1 | -.940^**^ | .332^**^ | .842^**^ | .767^**^ | .993^**^ | .300^**^ | .541^**^ | .278^**^ | -.034 | .303^**^ | .312^**^ | -.039 | .363^**^ |
|  | Sig. (2-tailed) | .000 | .365 | .000 | .000 | .000 |  | .000 | .000 | .000 | .000 | .000 | .001 | .000 | .003 | .725 | .001 | .001 | .687 | .000 |
|  | N | 110 | 110 | 110 | 110 | 110 | 110 | 110 | 110 | 110 | 110 | 110 | 110 | 110 | 110 | 110 | 110 | 110 | 110 | 110 |
| bio_07 | Pearson Correlation | -.827^**^ | -.014 | -.801^**^ | -.367^**^ | -.415^**^ | -.940^**^ | 1 | -.156 | -.804^**^ | -.511^**^ | -.921^**^ | -.307^**^ | -.550^**^ | -.308^**^ | .038 | -.319^**^ | -.326^**^ | -.003 | -.370^**^ |
|  | Sig. (2-tailed) | .000 | .885 | .000 | .000 | .000 | .000 |  | .104 | .000 | .000 | .000 | .001 | .000 | .001 | .695 | .001 | .000 | .978 | .000 |
|  | N | 110 | 110 | 110 | 110 | 110 | 110 | 110 | 110 | 110 | 110 | 110 | 110 | 110 | 110 | 110 | 110 | 110 | 110 | 110 |
| bio_08 | Pearson Correlation | .453^**^ | .218^*^ | .228^*^ | -.213^*^ | .561^**^ | .332^**^ | -.156 | 1 | .014 | .529^**^ | .360^**^ | -.240^*^ | .144 | -.336^**^ | .343^**^ | .047 | -.302^**^ | .193^*^ | -.309^**^ |
|  | Sig. (2-tailed) | .000 | .022 | .017 | .025 | .000 | .000 | .104 |  | .882 | .000 | .000 | .012 | .134 | .000 | .000 | .629 | .001 | .043 | .001 |
|  | N | 110 | 110 | 110 | 110 | 110 | 110 | 110 | 110 | 110 | 110 | 110 | 110 | 110 | 110 | 110 | 110 | 110 | 110 | 110 |
| bio_09 | Pearson Correlation | .803^**^ | .039 | .598^**^ | .373^**^ | .563^**^ | .842^**^ | -.804^**^ | .014 | 1 | .630^**^ | .837^**^ | .479^**^ | .550^**^ | .452^**^ | -.171 | .379^**^ | .477^**^ | -.073 | .578^**^ |
|  | Sig. (2-tailed) | .000 | .683 | .000 | .000 | .000 | .000 | .000 | .882 |  | .000 | .000 | .000 | .000 | .000 | .074 | .000 | .000 | .449 | .000 |
|  | N | 110 | 110 | 110 | 110 | 110 | 110 | 110 | 110 | 110 | 110 | 110 | 110 | 110 | 110 | 110 | 110 | 110 | 110 | 110 |
| bio_10 | Pearson Correlation | .887^**^ | .026 | .299^**^ | .303^**^ | .976^**^ | .767^**^ | -.511^**^ | .529^**^ | .630^**^ | 1 | .769^**^ | .258^**^ | .383^**^ | .191^*^ | -.074 | .229^*^ | .239^*^ | -.032 | .287^**^ |
|  | Sig. (2-tailed) | .000 | .788 | .002 | .001 | .000 | .000 | .000 | .000 | .000 |  | .000 | .006 | .000 | .045 | .442 | .016 | .012 | .741 | .002 |
|  | N | 110 | 110 | 110 | 110 | 110 | 110 | 110 | 110 | 110 | 110 | 110 | 110 | 110 | 110 | 110 | 110 | 110 | 110 | 110 |
| bio_11 | Pearson Correlation | .976^**^ | .188^*^ | .792^**^ | .315^**^ | .722^**^ | .993^**^ | -.921^**^ | .360^**^ | .837^**^ | .769^**^ | 1 | .246^**^ | .544^**^ | .215^*^ | .038 | .294^**^ | .249^**^ | -.055 | .308^**^ |
|  | Sig. (2-tailed) | .000 | .050 | .000 | .001 | .000 | .000 | .000 | .000 | .000 | .000 |  | .010 | .000 | .024 | .694 | .002 | .009 | .569 | .001 |
|  | N | 110 | 110 | 110 | 110 | 110 | 110 | 110 | 110 | 110 | 110 | 110 | 110 | 110 | 110 | 110 | 110 | 110 | 110 | 110 |
| bio_12 | Pearson Correlation | .236^*^ | -.310^**^ | -.050 | .589^**^ | .158 | .300^**^ | -.307^**^ | -.240^*^ | .479^**^ | .258^**^ | .246^**^ | 1 | .426^**^ | .889^**^ | -.737^**^ | .619^**^ | .906^**^ | .475^**^ | .928^**^ |
|  | Sig. (2-tailed) | .013 | .001 | .601 | .000 | .099 | .001 | .001 | .012 | .000 | .006 | .010 |  | .000 | .000 | .000 | .000 | .000 | .000 | .000 |
|  | N | 110 | 110 | 110 | 110 | 110 | 110 | 110 | 110 | 110 | 110 | 110 | 110 | 110 | 110 | 110 | 110 | 110 | 110 | 110 |
| bio_13 | Pearson Correlation | .526^**^ | -.079 | .422^**^ | .024 | .293^**^ | .541^**^ | -.550^**^ | .144 | .550^**^ | .383^**^ | .544^**^ | .426^**^ | 1 | .105 | .238^*^ | .893^**^ | .122 | .568^**^ | .238^*^ |
|  | Sig. (2-tailed) | .000 | .414 | .000 | .801 | .002 | .000 | .000 | .134 | .000 | .000 | .000 | .000 |  | .276 | .012 | .000 | .205 | .000 | .012 |
|  | N | 110 | 110 | 110 | 110 | 110 | 110 | 110 | 110 | 110 | 110 | 110 | 110 | 110 | 110 | 110 | 110 | 110 | 110 | 110 |
| bio_14 | Pearson Correlation | .182 | -.308^**^ | -.067 | .734^**^ | .098 | .278^**^ | -.308^**^ | -.336^**^ | .452^**^ | .191^*^ | .215^*^ | .889^**^ | .105 | 1 | -.904^**^ | .253^**^ | .991^**^ | .161 | .961^**^ |
|  | Sig. (2-tailed) | .056 | .001 | .484 | .000 | .307 | .003 | .001 | .000 | .000 | .045 | .024 | .000 | .276 |  | .000 | .008 | .000 | .092 | .000 |
|  | N | 110 | 110 | 110 | 110 | 110 | 110 | 110 | 110 | 110 | 110 | 110 | 110 | 110 | 110 | 110 | 110 | 110 | 110 | 110 |
| bio_15 | Pearson Correlation | .039 | .364^**^ | .329^**^ | -.732^**^ | -.011 | -.034 | .038 | .343^**^ | -.171 | -.074 | .038 | -.737^**^ | .238^*^ | -.904^**^ | 1 | .044 | -.906^**^ | .010 | -.834^**^ |
|  | Sig. (2-tailed) | .686 | .000 | .000 | .000 | .906 | .725 | .695 | .000 | .074 | .442 | .694 | .000 | .012 | .000 |  | .650 | .000 | .918 | .000 |
|  | N | 110 | 110 | 110 | 110 | 110 | 110 | 110 | 110 | 110 | 110 | 110 | 110 | 110 | 110 | 110 | 110 | 110 | 110 | 110 |
| bio_16 | Pearson Correlation | .289^**^ | -.155 | .172 | .038 | .139 | .303^**^ | -.319^**^ | .047 | .379^**^ | .229^*^ | .294^**^ | .619^**^ | .893^**^ | .253^**^ | .044 | 1 | .269^**^ | .806^**^ | .366^**^ |
|  | Sig. (2-tailed) | .002 | .106 | .073 | .695 | .148 | .001 | .001 | .629 | .000 | .016 | .002 | .000 | .000 | .008 | .650 |  | .005 | .000 | .000 |
|  | N | 110 | 110 | 110 | 110 | 110 | 110 | 110 | 110 | 110 | 110 | 110 | 110 | 110 | 110 | 110 | 110 | 110 | 110 | 110 |
| bio_17 | Pearson Correlation | .225^*^ | -.303^**^ | -.055 | .735^**^ | .148 | .312^**^ | -.326^**^ | -.302^**^ | .477^**^ | .239^*^ | .249^**^ | .906^**^ | .122 | .991^**^ | -.906^**^ | .269^**^ | 1 | .162 | .976^**^ |
|  | Sig. (2-tailed) | .018 | .001 | .568 | .000 | .123 | .001 | .000 | .001 | .000 | .012 | .009 | .000 | .205 | .000 | .000 | .005 |  | .091 | .000 |
|  | N | 110 | 110 | 110 | 110 | 110 | 110 | 110 | 110 | 110 | 110 | 110 | 110 | 110 | 110 | 110 | 110 | 110 | 110 | 110 |
| bio_18 | Pearson Correlation | -.050 | -.191^*^ | -.091 | -.109 | -.109 | -.039 | -.003 | .193^*^ | -.073 | -.032 | -.055 | .475^**^ | .568^**^ | .161 | .010 | .806^**^ | .162 | 1 | .186 |
|  | Sig. (2-tailed) | .604 | .046 | .346 | .256 | .255 | .687 | .978 | .043 | .449 | .741 | .569 | .000 | .000 | .092 | .918 | .000 | .091 |  | .052 |
|  | N | 110 | 110 | 110 | 110 | 110 | 110 | 110 | 110 | 110 | 110 | 110 | 110 | 110 | 110 | 110 | 110 | 110 | 110 | 110 |
| bio_19 | Pearson Correlation | .286^**^ | -.271^**^ | .002 | .684^**^ | .194^*^ | .363^**^ | -.370^**^ | -.309^**^ | .578^**^ | .287^**^ | .308^**^ | .928^**^ | .238^*^ | .961^**^ | -.834^**^ | .366^**^ | .976^**^ | .186 | 1 |
|  | Sig. (2-tailed) | .002 | .004 | .980 | .000 | .043 | .000 | .000 | .001 | .000 | .002 | .001 | .000 | .012 | .000 | .000 | .000 | .000 | .052 |  |
|  | N | 110 | 110 | 110 | 110 | 110 | 110 | 110 | 110 | 110 | 110 | 110 | 110 | 110 | 110 | 110 | 110 | 110 | 110 | 110 |
| **. Correlation is significant at the 0.01 level (2-tailed). | | | | | | | | | | | | | | | | | | | | |
| *. Correlation is significant at the 0.05 level (2-tailed). | | | | | | | | | | | | | | | | | | | | |
